# Chronic hypoxia in pregnancy affected vascular tone of renal interlobar arteries in the offspring

**DOI:** 10.1038/srep09723

**Published:** 2015-05-18

**Authors:** Jiaqi Tang, Zhoufeng Zhu, Shuixiu Xia, Na Li, Ningjing Chen, Qinqin Gao, Lingjun Li, Xiuwen Zhou, Dawei Li, Xiaolin Zhu, Qing Tu, Weisheng Li, Chonglong Wu, Jiayue Li, Yuan Zhong, Xiang Li, Caiping Mao, Zhice Xu

**Affiliations:** 1 Institute for Fetology, First Hospital of Soochow University, Suzhou, China; 2 Center for Perinatal Biology, Loma Linda University, California, USA

## Abstract

Hypoxia during pregnancy could affect development of fetuses as well as cardiovascular systems in the offspring. This study was the first to demonstrate the influence and related mechanisms of prenatal hypoxia (PH) on renal interlobar arteries (RIA) in the 5-month-old male rat offspring. Following chronic hypoxia during pregnancy, phenylephrine induced significantly higher pressor responses and greater vasoconstrictions in the offspring. Nitric oxide mediated vessel relaxation was altered in the RIA. Phenylephrine-stimulated free intracellular calcium was significantly higher in the RIA of the PH group. The activity and expression of L-type calcium channel (Cav1.2), not T-type calcium channel (Cav3.2), was up-regulated. The whole-cell currents of calcium channels and the currents of Cav1.2 were increased compared with the control. In addition, the whole-cell K^+^ currents were decreased in the offspring exposed to prenatal hypoxia. Activity of large-conductance Ca^2+^-activated K^+^ channels and the expression of MaxiKα was decreased in the PH group. The results provide new information regarding the influence of prenatal hypoxia on the development of the renal vascular system, and possible underlying cellular and ion channel mechanisms involved.

Renal vascular systems play a vital role in the regulation of vascular volume. Recent studies demonstrated that adverse factors during pregnancy could cause poor renal development in the fetus and offspring^1^,^2^. Hypoxia *in utero* not only altered renal development in the fetuses^3^, but also induced programming of aberrant kidney development and accelerate the aging process of the kidney during postnatal development^4^,^5^. Our recent work showed that exposure to hypoxia during pregnancy caused Becn1 signaling-mediated renal autophagy in the rat fetuses^3^. Autophagy is type II programmed cell death, which is different from type I programmed cell death apoptosis. Both of them can cause structural changes in cells^6^,^7^,^8^. However, it is unknown whether autophagy or apoptosis may occur in renal vascular systems following chronic hypoxia during early developmental periods. Addressing such questions is very important to further understand the etiology and pathology of renal and vascular diseases in developmental origins. Thus, this study used the prenatal hypoxia (PH) model and determined key molecular elements in the pathways of autophagy and apoptosis in the renal vessels of the offspring exposed to chronic hypoxia during pregnancy.

A number of adverse environmental factors (e.g. nicotine, malnutrition, and cocaine, etc.) during prenatal stage could induce vascular problems and increase susceptibility of cardiovascular diseases^9^,^10^,^11^,^12^,^13^. *In utero* hypoxia could contribute to an increased risk of cardiovascular diseases^4^ and altered vascular functions^14^. The present study hypothesized that prenatal hypoxia may induce functional and molecular changes in renal blood vessels.

Altered vascular functions could result from either enhanced vasoconstriction or reduced vessel relaxation. For example, the offspring from hypoxic pregnancy had markedly impaired nitric oxide-dependent relaxation in peripheral resistance arteries^15^,^16^. Voltage-gated Ca^2+^ channels play a central role in the regulation of intracellular calcium ([Ca^2+^]i) and vascular tone^17^. In the present study, we measured vascular relaxation and [Ca^2+^]i in the renal vessels of the offspring. In addition, functions and expression of L-type (Cav1.2) and T-type calcium channel (Cav3.2) were tested.

Potassium channels play critical roles in the regulation of smooth muscle membrane potential and thereby controlling smooth muscle tone^18^. Large-conductance Ca^2+^-activated K^+^ channels (BK), one of the important K^+^ channels, are dominantly expressed in vascular smooth muscle cells^19^,^20^. Notably, there has been very limited information regarding influence and underlying mechanism of prenatal hypoxia-affected ion channel activity in renal vessels. The present study also determined functions and expression of BK. Together, the data gained may provide important information on whether and how chronic hypoxia during early developmental stages affects renal blood vessels.

## Results

### Kidney and body weight

**Figure 1:**
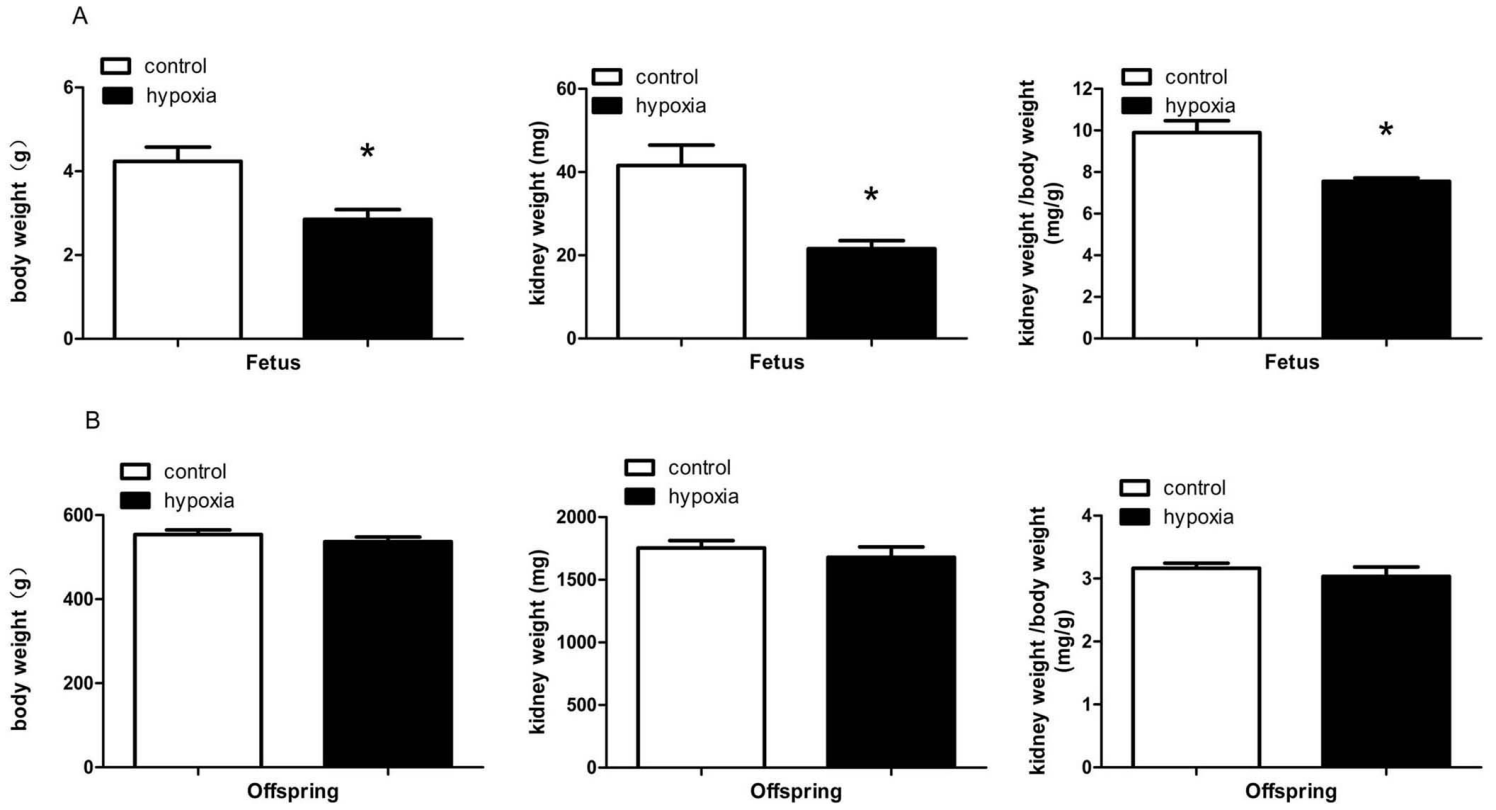
The effect of prenatal hypoxia on body weight, kidney weight, ratio of kidney/body weight in fetuses and 5-month-old offspring. *,P < 0.05; N = 10.

Prenatal hypoxia resulted in a significant decrease in body weight, kidney weight, and the ratio of kidney-to-body weight in the fetuses at gestational day 21 (P < 0.05; Figure 1A). For 5-month-old offspring, there were no significant differences in body and kidney weight as well as the ratio of kidney-to-body weight between the control and PH group (P > 0.05; Figure 1B).

### ELISA analysis

**Figure 2:**
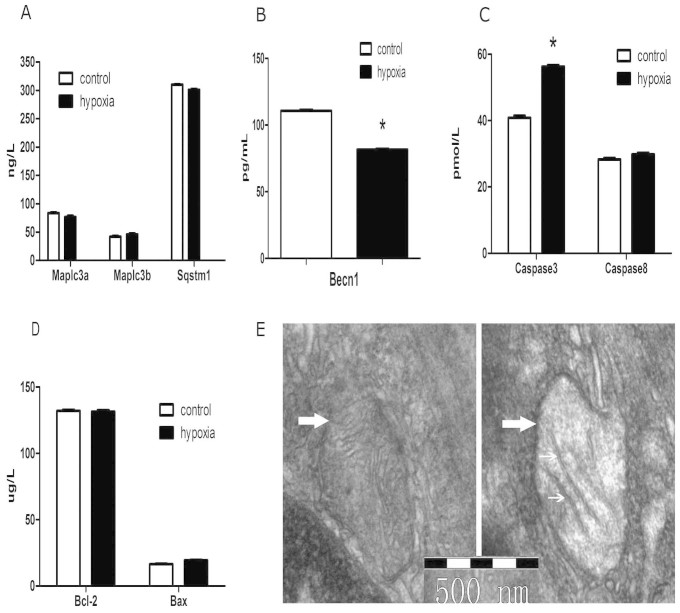
(A–D) Expression of Becn1, Map1lc3a, Map1lc3b, Sqstm1, Caspase3, Bax, Bcl2, and Caspase8 in renal interlobar arteries of 5-month-old offspring. *,P < 0.05; N = 7. (E) Transmission electron microscope analysis on smooth muscle cells of offspring renal arteries. Thick arrow: mitochondrion. The aberrant phenomenon (the lines in the mitochondrion indicated by thin arrow) showed mitochondrion swelling (Left: control, right: PH).

The expression of Becn1 was significantly reduced in renal interlobar arteries of the PH offspring at 5 months old, where Caspase3 was increased (P < 0.05; Figure 2B–C). Map1lc3a, Map1lc3b, Sqstm1, Bax, Bcl2, and Caspase8 were not significantly different in renal arteries between the control and PH group (P > 0.05; Figure 2A, C, D).

### Transmission electron microscope analysis

Transmission electron microscope analysis showed that significant mitochondrial injury in the hypoxia offspring characterized by mitochondrial swelling and disappearance of mitochondrial cristae in vascular smooth muscle cells of renal interlobar arteries (Figure 2E).

### Blood pressure

**Figure 3:**
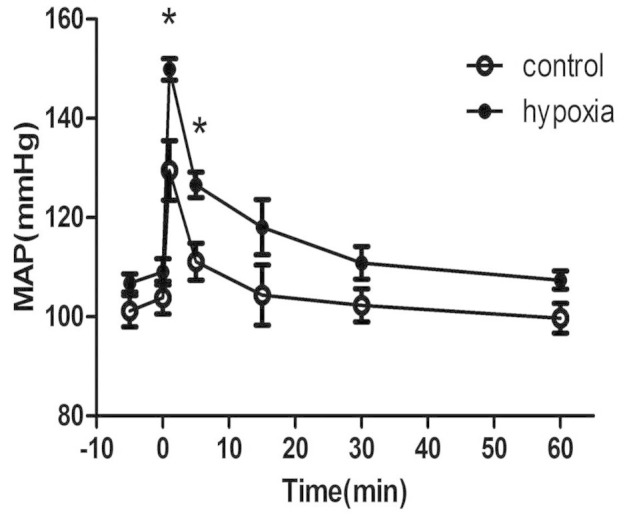
Phenylephrine increased mean arterial pressure (MAP) in free moving offspring. 0 minute: injection time. Hypoxia: prenatal hypoxia. *,P < 0.05; N = 5.

Following administration of phenylephrine, mean arterial pressure (MAP) in the PH offspring was significantly higher than that in the control (P < 0.05; Figure 3).

### Vascular tension in renal interlobar arteries

**Figure 4:**
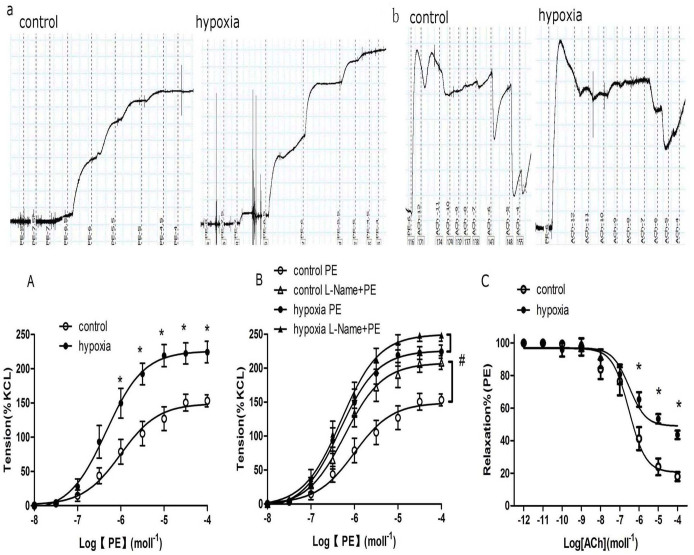
(a) Representative images of phenylephrine-mediated vasoconstrictions in renal interlobar arteries of the control and hypoxia offspring. (b) Representative images of vasodilatation by acetylcholine based on phenylephrine-increased vascular tension. (A) The effect of chronic hypoxia during pregnancy on vascular responses to phenylephrine (PE) in offspring renal interlobar arteries. *,P < 0.05; N = 8. (B) PE-increased vasoconstriction in presence or absence of NG-Nitro-l-arginine (L-Name). #,P < 0.05; N = 6. (C) Acetylcholine (ACh)-mediated vasodilatation. The arteries were contracted with PE (1 μmoll^−1^) prior to addition of ACh. *,P < 0.05; N = 5–6.

There was an increase in phenylephrine-mediated vascular tension in the renal interlobar arteries. Phenylephrine-induced maximum contractions were significantly higher in the PH offspring than that in the control (P < 0.05; Figure 4A). There was a significant difference in response to PE between the control and PH offspring in the absence or presence of NG-Nitro-l-arginine (L-Name, nitric oxide-synthase inhibitor) (Figure 4B). Acetylcholine (ACh)-mediated relaxation was weaker in the PH offspring (Figure 4C). Vessel integrity was confirmed immediately after the experiments using histologic analysis.

**Figure 5:**
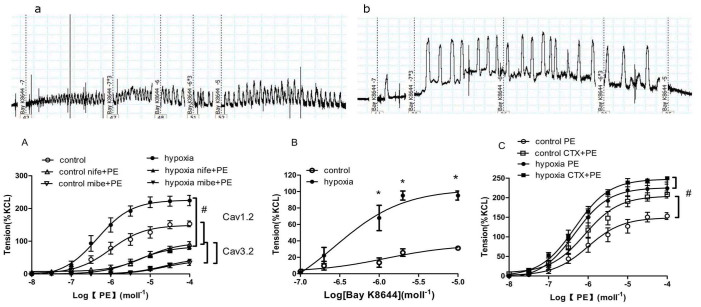
(a, b): Representative images of Bay K8644-mediated vasoconstrictions, (a): control, (b): prenatal hypoxia. The distance between two adjacent transverse lines represents 0.5 mN. (A) The effect of mibefradil (mibe), nifedipine (nife) on phenylephrine (PE)-mediated vasoconstrictions respectively in offspring renal interlobar arteries. #,P < 0.05; N = 6. (B) Vessel tension to Bay K8644 in renal interlobar arteries. *,P < 0.05; N = 5. (C) The effect of charybdotoxin (CTX) on PE-mediated vasoconstrictions. #,P < 0.05; N = 7.

Mibefradil (a blocker for Cav1.2 and Cav3.2 channels) and nifedipine (a specific Cav1.2 channel blocker) depressed PE–induced contractile responses, and the decreased levels in the PH offspring were significantly greater than that in the control (Figure 5A). Figure 5A showed that PE-mediated vessel tension was reduced to the same levels between the two groups by either mibefradil or nifedipine. Bay K8644 (an agonist for voltage-dependent calcium channel) caused stronger contractions in the renal interlobar arteries in the PH offspring than that in the control (Figure 5B). In both groups, CTX (a BK channel blocker) potentiated PE-induced contractions; the increased level was greater in the control offspring (Figure 5C).

### [Ca^2+^]i in renal interlobar arteries

**Figure 6:**
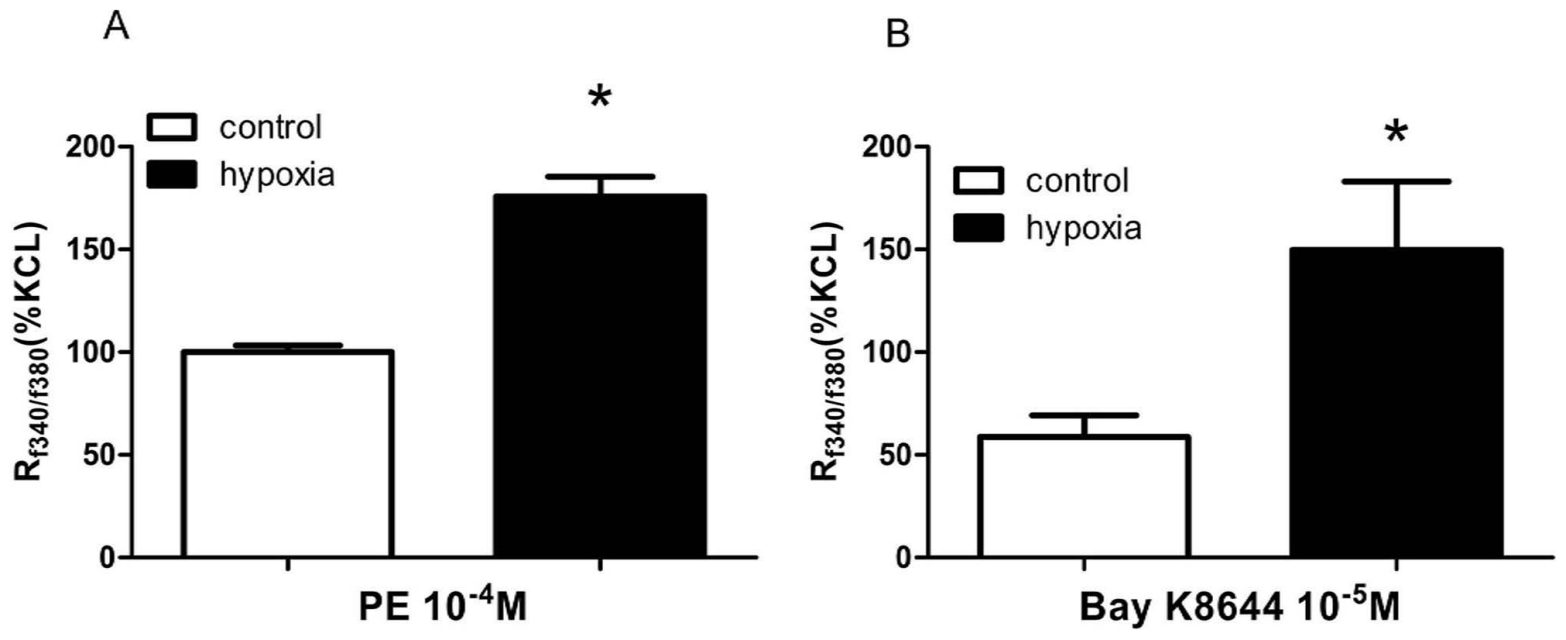
Free intracellular calcium ([Ca^2+^]i) in response to phenylephrine (PE) 10^−4^ M (A) and Bay K8644 10^−5^ M (B) in renal interlobar arteries. *,P < 0.05; N = 6–7.

Phenylephrine and Bay k8644 mediated-increase of [Ca^2+^]i was significantly higher in renal interlobar arteries of the PH group (P < 0.05; Figure 6).

### Whole-cell Ca^2+^ currents and K^+^ currents on vascular smooth muscle cells

**Figure 7:**
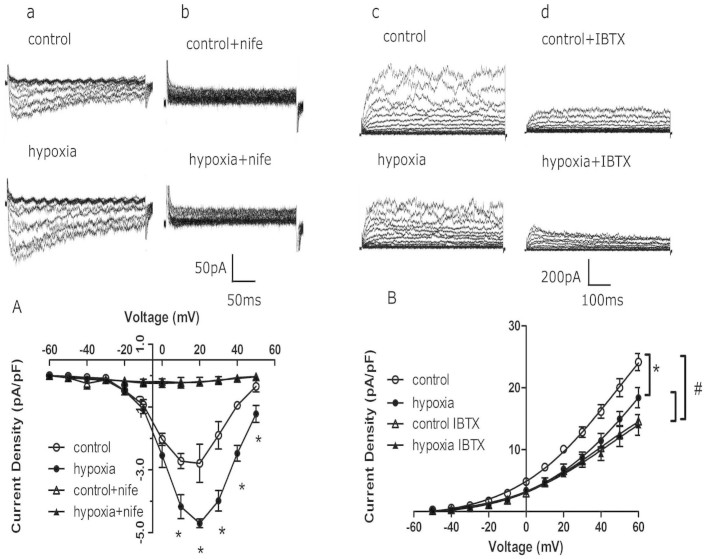
The effect of prenatal hypoxia on calcium currents and K^+^ currents in renal vascular smooth muscle cells (VSMCs) of offspring. (a, b): images of whole-cell calcium currents in absence or presence of nifedipine in both groups, (c, d): images of whole-cell potassium channel currents in the absence or presence of iberiotoxin (IBTX). (A) Calcium current traces recorded in renal VSMCs in the absence or presence of nifedipine (nife). *,P < 0.05; N = 3–6. (B) K^+^ current traces recorded in VSMCs in the absence or presence of iberiotoxin (IBTX). #,P < 0.05; N = 4–10.

The calcium current amplitude was significantly higher in the PH vascular smooth muscle cells (VSMCs) from renal interlobar arteries than that in the control (P < 0.05), and the maximum current density calculated at +20 mV in the PH VSMCs was remarkably increased compared with the control. Incubation with nifedipine depressed Ca^2+^ current density in both groups, while the decreased level was greater in the PH group (Figure 7A). Whole-cell K^+^ current density tested from −10 to +60 mV in the PH VSMCs was significantly depressed compared with that in the control, the maximum current density calculated at +60 mV was 24.1638 ± 1.3818 pA/pF in the control and 18.3982 ± 2.6347 pA/pF in the PH (P < 0.05; Figure 7B). Exposure to 100 nmoll^−1^ iberiotoxin (IBTX, a selective and reversible inhibitor of BK channel) significantly depressed K^+^ currents. I–V relationship displayed that whole-cell K^+^ current density at +60 mV inhibited by IBTX in the VSMCs was 39.54% in the control, and 23.89% in the PH, respectively (Figure 7B).

### Western blot analysis

**Figure 8:**
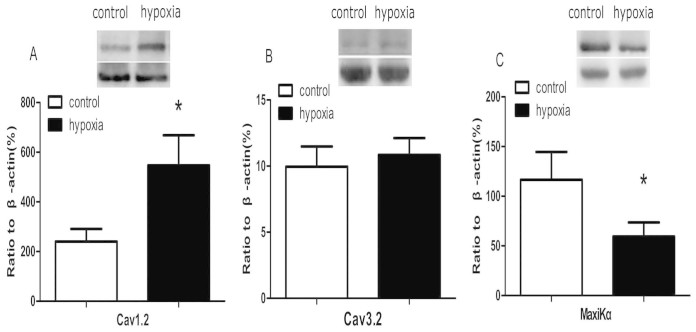
The expression of Cav1.2α1C, Cav3.2α1H, and MaxiKα in offspring renal interlobar arteries. *,P < 0.05; N = 7.

Cav1.2α1C was significantly higher in renal interlobar arteries of the PH (P < 0.05; Figure 8A). There was no difference in Cav3.2α1H between the control and PH offspring (P > 0.05; Figure 8B). Western blot analysis showed a significant reduction in the expression of MaxiKα protein in the PH offspring (P < 0.05; Figure 8C).

## Discussion

Multiple adverse factors during pregnancy may cause poor renal development in fetuses and influence health of the offspring^1^,^2^,^3^. Hypoxia *in utero* during the critical developmental periods has been demonstrated to cause programming of aberrant renal development and accelerate aging process of the kidney^4^,^21^. In the present study, prenatal hypoxia resulted in a decrease in fetal kidney and body weight as well as the ratio of kidney/body weight, indicating that renal development was affected by the hypoxia. However, those weight differences disappeared in the adult offspring, probably due to “catch-up” growth as previously reported^22^.

A recent study demonstrated that similar prenatal exposure to hypoxia altered renal development in the rat fetuses and induced Becn1 signaling-mediated renal autophagy in the renal tissue by using multiple approaches, including measuring of protein, mRNA, and immunostaining^3^. In the present study, Becn1 expression was significantly decreased, while caspase3 was increased in the renal blood vessels of the PH offspring, although Map1lc3a, Map1lc3b, Sqstm1, Bax, Bcl2, and caspase8 were not changed. The changes in the expression of Becn1 and caspase3 in the RIA suggest that both autophagy (type II cell death) and apoptosis (type I cell death) may occur in the renal blood vessels following prenatal hypoxia, adding new information on prenatal hypoxia-affected development in kidney vascular systems. Further histological analysis showed significant mitochondrial swelling in vascular smooth muscle cells of the renal arteries in the hypoxia offspring. This histological alteration was associated with molecular changes of Becn1 and caspase3 in the RIA, indicating a possible link between mitochondrial injury and cellular death, and certain molecular and mitochondrial changes in the renal blood vessels following prenatal hypoxia.

Whether renal blood vessels can be functionally influenced by prenatal factors is an important question and the focus of the present study, since accumulated evidence have suggested a relationship between environmental insults during prenatal periods and increased cardiovascular risks in postnatal life^2^,^9^,^11^,^13^. The present study found PE-mediated pressor responses and vascular tension of RIA were significantly higher in the PH group, as new finding of functional changes in blood pressure and renal vascular responses following prenatal exposure to hypoxia. Increased vascular tension by PE could result from either enhanced vessel contraction or reduced relaxation. Previous studies showed a markedly impaired nitric oxide-dependent relaxation in mesenteric arteries and femoral arteries in the offspring exposed to prenatal hypoxia^15^,^16^. Nitric oxide mediation of endothelium-dependent relaxation was evaluated using L-Name and acetylcholine (ACh) in the present study. L-Name significantly potentiated PE-mediated vasoconstrictions in both groups; however, the potentiated effect by L-Name was significantly weaker in the PH than that in the control group. Acetylcholine acts on cholinergic receptors in blood vessels, by stimulating the endothelium to produce NO that causes vascular relaxation. The present study demonstrated that ACh-induced relaxation was significantly decreased in the PH offspring. These results indicated that NO functions or pathways in the endothelium of renal interlobar arteries might be injured in the PH offspring, which could cause reduced vascular relaxation. To best of our knowledge, this was the first in demonstration of renal vascular dysfunctions following chronic exposure to adverse environments during pregnancy. Small arteries in the kidney, especially afferent arterioles, play important roles in renal autoregulation^23^. The present study found functional and molecular changes in the interlobar arteries, which suggested that afferent arterioles in the same kidney could be affected too. This is worth further investigation.

Phenylephrine is a widely accepted vasoconstrictor. One of underlying mechanisms for PE-mediated vasoconstrictions is linked to free intracellular calcium. A rapid increase in [Ca^2+^]i is a major determinant of vascular contraction. Ca^2+^ binds to calmodulin causing contraction in smooth muscle cells^24^,^25^. In the present study, PE significantly increased [Ca^2+^]i in renal interlobar arteries. Notably, the increased levels of [Ca^2+^]i was higher in the PH offspring than that in the control. In addition, vasoconstrictions and [Ca^2+^]i were greater by Bay K8644 in renal interlobar arteries of the PH offspring. Moreover, patch clamp experiments revealed that whole-cell calcium currents were significantly higher in the smooth muscle cells from the PH offspring. Together, those results provided new information that voltage-dependent calcium channels played an important role in the PE-increased renal vascular tension in the PH group.

Calcium influx through voltage dependent calcium channel is an important pathway for the regulation of [Ca^2+^]i^17^,^26^. Both Cav1.2 (L- type calcium channel) and Cav3.2 (T- type calcium channel) are involved in [Ca^2+^]i-mediated vasoconstriction^27^. Expression levels of Cav1.2 and Cav3.2 in the intrarenal arteries are dominant^28^. One previous study demonstrated that chronic hypoxia selectively enhanced both L- and T-type voltage-dependent Ca^2+^ channel activity in the pulmonary artery by up-regulating Cav1.2 and Cav3.2^27^. In the present study, we tested both Cav1.2 and Cav3.2 in renal interlobar arteries of the offspring. The data showed that the pretreatment with either mibefradil or nifedipine could significantly reduce PE-increased vascular tension, and the inhibition by either mibefradil or nifedipine was greater in the PH than that in the control offspring. In addition, the inhibition by mibefradil was stronger than that by nifedipine in both groups. These results suggested that L-type voltage-dependent Ca^2+^ channels, not T-type, played critical roles in the PE-increased vasoconstrictions via [Ca^2+^]i in the renal blood vessels of the PH offspring. To further determine the possible influence of L-type or T-type channels, we did patch clamp and protein analysis. The results showed that Cav1.2 currents were significantly increased in the smooth muscle cells, in association with significantly higher expression of Cav1.2α1C in renal arteries of the PH offspring, which may contribute to the increased [Ca^2+^]i and vascular tension by PE in the PH offspring.

Besides calcium channels, K^+^ channels also are critical for vascular functions. Potassium channels play roles in regulating VSMC membrane potentials, and thereby controlling vascular tone^18^. In the present study, charybotoxin potentiated-vasoconstriction by PE was altered in the PH offspring compared with the control, indicating K^+^ channels in renal interlobar arteries were affected, which could contribute to the increase of the stimulated vascular tension in the kidney of the PH offspring. Furthermore, BK current density in smooth muscle cells from renal arteries was decreased by IBTX in the PH offspring. One of important K^+^ channels is BK channels that exist in vascular muscle cell^18^,^19^. BK channels appear to play a negative feedback role in limiting active vasoconstrictions^29^, as well as in development of hypertension^30^. The expression of BK channels in vascular smooth muscle membranes was increased in hypertension^19^. In the present study, BK functions and BK current density in smooth muscle cells from renal arteries were decreased in the PH offspring. The expression of MaxiKα protein in the renal vessels was also reduced in the PH offspring. This was the first to demonstrate that the prenatal factor could affect BK channels in renal arteries of the offspring. Those data provide new information that prenatal insults such as chronic hypoxia could induce molecular changes in expression of BK protein and alter functions of BK channels in the renal arteries.

## Conclusions

The present study demonstrated that prenatal hypoxia had significant influence on PE-mediated blood pressure, histological structures, molecular expression, vascular tissue functions, and ion channel activity in smooth muscle cells and in renal arteries of the offspring. Importantly, PE-stimulated pressor responses and renal vascular tension were increased by prenatal hypoxia, which could be critical contribution to vascular diseases in life later. To determine possible underlying mechanisms, the present study found both vascular abilities in relaxation and constriction were changed following exposure to chronic prenatal hypoxia. Those findings include: NO functions in renal arteries were disabled, PE-increased free intracellular calcium was higher, and the whole-cell K^+^ currents, especially BK, were relatively weaker in the renal vascular tissue. Subsequent testing found the probable causes for the observed phenomenon in altered L-type voltage-dependent Ca^2+^ channel activity, which included the changes in Cav1.2 currents and protein expression, altered BK channel currents and reduced MaxiKα expression in the renal vessels and smooth muscle cells. Together, the data gained provide important information to further understand the long-term influence and mechanisms of chronic hypoxia during early developmental periods on the renal vascular system, which may offer new insight for developing new preventive medicines or approaches targeting at vascular and cellular levels against renal and vascular diseases in fetal origins.

## Methods

### Animals

Pregnant Sprague-Dawley rats from the Animal Center of Soochow University were randomly divided into the control (21% oxygen) and hypoxia group (10.5% oxygen) from gestational day 4 to 21. Hypoxia was induced by a mixture of nitrogen gas and air in an individual chamber. The control group was housed with room air flowing through chambers. All animals were fed with standard food and tap water. Some of the pregnant rats were sacrificed on day 21 of gestation for fetal studies. Others were allowed to give birth naturally. Pups were kept with their mothers until weaning. The male offspring were tested at 5 months old. Kidneys were collected immediately after sacrifice of animals. Body and kidney weight was determined. All experimental procedures were approved by the Institutional Animal Care Committee and in accordance with the Guide for the Care and Use of Laboratory Animals (NIH Publication No. 85–23, 1996).

### ELISA and transmission electron microscope analysis

The contents of Map1lc3a, Map1lc3b, Sqstm1, Becn1, Caspase3, Caspase8, Bax, and Bcl2 in RIA tissue of the offspring were measured with enzyme-linked immunosorbent assay (ELISA) using commercially available kits by Y-Y Biochemical Corporation (Shanghai, China). All data were processed in a blind manner.

Renal arteries of 5 month-old offspring were isolated and fixed in 4% paraformaldehyde at 4°C, and then were prepared and examined using transmission electron microscope (JEOL-1010, Japan) in a blind manner at Nanjing Medical University.

### Measurement of blood pressure

Rats were anesthetized with a mixture of ketamine (75 mg/kg) and xylazine (10 mg/kg) (Hengrui Medicine, Jiangsu, China) intraperitoneally. Polyethylene catheters were placed in the femoral artery and threaded to the abdominal aorta^8^,^11^. After three days of recovery from vascular catheterization, blood pressure was recorded via the artery catheter in conscious and freely moving offspring rats. At testing, phenylephrine (10 μg/kg) was injected via the catheter, blood pressure was recorded for at least 60 minutes using Power Lab system and software (AD Instruments, Australia).

### Measurement of vascular tension in renal interlobar arteries

Rats were sacrificed using sodium pentobarbital (100 mg/kg; Hengrui Medicine, Jiangsu, China) intraperitoneally. RIA was dissected from connective tissues and cut into spiral rings. Isolated rings (3–4 mm in length) were suspended in organ chambers filled with HEPES-PSS solution (mmoll^−1^; NaCL 141.85, KCL 4.7, MgSO_4_ 1.7, EDTA 0.51, CaCl_2_.2H_2_O 2.79, KH2PO4 1.17, glucose 5.0 and HEPES 10.0; pH7.4), gassed continuously with 95% O2–5% CO2. Wire myograph (Dual Wire Myograph System, Model 410A; DMTA/S, Aarhus, Denmark) was used to measure vasoconstrictions in RIA from offspring. Potassium chloride (120 mmoll^−1^) was used to achieve optimal resting tension before adding drugs. Vasoconstrictions induced by the drugs were normalized by comparing with the contraction elicited by KCL alone. Induced vasoconstrictions were obtained following cumulative addition of phenylephrine (PE, 10^−8^–10^−4^ moll^−1^). NG-Nitro-l-arginine (L-Name, nitric oxide-synthase inhibitor; 100 μmoll^−1^), mibefradil (antagonist for Cav1.2 and Cav3.2; 10 μmoll^−1^), or nifedipine(antagonist for Cav1.2; 1 μmoll^−1^)was added into the chambers for 30 minutes before application of PE. Acetylcholine (10^−12^–10^−4^ moll^−1^) was used following application of PE (1 μmoll^−1^) that produced and maintained vasoconstrictions at a steady state for at least 15–20 min. Bay K8644 (agonist for voltage dependent calcium channels, 10^−7^–10^−5^ moll^−1^) was used for testing dose-related contractions. After pre-treating vessel rings with charybdotoxin (CTX, BK channel antagonist, 100 nmoll^−1^)for 2 hours, PE-mediated contraction was tested. All drugs were purchased from Sigma (St. Louis, USA).

### Measurement of intracellular Ca^2+^ in renal interlobar arteries

In the RIA, [Ca^2+^]i concentrations in vascular tissue were measured using fluorescent Ca^2+^ indicator, the acetoxymethyl ester of fura-2 (fura2-AM; Calbiochem, San Diego, CA, USA). In brief, RIA ring was loaded in HEPES-PSS solution with fura2-AM (10 μmoll^−1^) for 4 hours at room temperature, gassed continuously with 95% O2–5% CO2. Then rings were incubated in HEPES-PSS solution for 30 minutes at 37°C. [Ca^2+^]i levels were calculated qualitatively by fluorescence ratio of fura-2AM at 340 and 380 nm wave length (Rf340/380). PE (10^−4^ moll^−1^) and Bay K8644 (10^−5^ moll^−1^) was added to the chamber respectively, and vessel responses were monitored and recorded continuously.

### Electrophysiological measurement

Vascular smooth muscle cells were isolated enzymatically from dissected RIA as reported^11^,^31^. Whole-cell Ca^2+^ channel currents and whole cell K^+^ currents were recorded in conventional whole-cell configuration, voltage-clamp mode using an Axon Multiclamp 700B with Clampex10.1 and normalized to cell capacitance as picoampere per picofarad (pA/pF). Voltage-dependent Ca^2+^ channel current densities were assessed using standard pulse protocols and a patch-clamp station described previously^11^,^32^. Negative inward current through Cav1.2 was evaluated using Ba^2+^ as the charge carrier. T-type Ca^2+^ current was eliminated by using a holding potential of −60 mV. IBa was evoked using several voltage protocols. For activation of the inward current, Ba^2+^ current was elicited by 250 ms voltage steps from holding potential of −60 mV to test potentials in the range from −60 to 50 mV with 10 mV increments. Barium chloride (10 mmoll^−1^) as the charge carrier was used to limit current rundown. In a subset of cells, 1 μmoll^−1^ nifedipine was used to verify the identity of Cav1.2 currents. Only cells with an input resistance >2 GΩ without substantial run-down were analyzed. Whole cell K^+^ currents were elicited by 500 ms voltage steps from holding potential of −70 mV to test potentials in the range from −60 to 60 mV with 10 mV increments. BK currents were tested with IBTX (100 nmoll^−1^).

### Western blot

Vascular tissue collected was homogenized. The homogenate containing 100 μg protein was quantified and loaded in each lane for electrophoresis, and then was transferred to immobilon polyvinyldifluoride membranes (Millipore, MA). Membranes were incubated overnight at 4°C with primary antibodies against Cav1.2 (1:200), Cav3.2 (1:200), or MaxiKα (BK α-subunits, 1:500)(Santa Cruz Biotech, Santa Cruz, CA), then incubated with donkey anti-goat antibody or goat anti-rabbit antibody (1:4,000), respectively, and visualized using chemiluminescence detection (Amersham Biosci., Piscataway, NJ) and UVP imaging system (EC3-Imaging-System, Upland, CA). Imaging signals were digitized and analyzed; the ratio of band intensity to β-actin was obtained for analysis.

### Data analysis

Concentration-response curves for vascular responses were analyzed by computer-assisted nonlinear regression (Graph Pad Prism software) to fit the data. Data was expressed as mean ± SEM. Statistical significance (P < 0.05) was determined by two-way ANOVA or t-test.

## Author Contributions

J.T., C.M. and Z.X. wrote the main manuscript text, S.X. and Q.T. determined physiological testing and figure 1, L.L. examined cell structures and prepared figure 2A–D. X.L. and Xiu. Z. did data analysis and prepared figure 2E. N.C. and Q.G. did blood pressure and vasodilatation to acetylcholine, and prepared figure 3 and 4C. J.T., Y.Z. and J.L. did vascular experiments and prepared figure 4–5, D.L. and C.W. measured intracellular Ca^2+^ and prepared figure 6, Z.Z. and N.L. did electrophysiological experiments and prepared figure 7, Xiao. Z. and W.L. did western blot and prepared figure 8. All authors reviewed the manuscript.

## Additional Information

**How to cite this article**: Tang, J. *et al.* Chronic hypoxia in pregnancy affected vascular tone of renal interlobar arteries in the offspring. *Sci. Rep.* 5, 9723; DOI:10.1038/srep09723 (2015).
